# First *De Novo* Draft Genome Sequence of the Pathogenic Fungus Fusarium udum F02845, Associated with Pigeonpea (Cajanus cajan L. Millspaugh) Wilt

**DOI:** 10.1128/MRA.01001-18

**Published:** 2018-10-04

**Authors:** Alok Kumar Srivastava, Prem Lal Kashyap, Hillol Chakdar, Murugan Kumar, Anchal Kumar Srivastava, Jagriti Yadav, Hena Jamali, Ruchi Srivastava, Anjney Sharma, Praveen Tiwari, Alok Singh, Anil Kumar Saxena

**Affiliations:** a ICAR-National Bureau of Agriculturally Important Microorganisms (NBAIM), Mau, Uttar Pradesh, India; b ICAR-Indian Institute of Wheat and Barley Research (IIWBR), Karnal, Haryana, India; Vanderbilt University

## Abstract

Fusarium udum F02845 is a destructive fungal pathogen which causes pigeonpea (Cajanus cajan L. Millspaugh) wilt. Here we report the first *de novo* draft assembly of Fusarium udum F02845, isolated from an infected pigeonpea stem. The genome was determined to be 56.38 Mb in size, with a G+C content of 42.44%, and predicted to have 712 scaffolds with a total number of 11,829 genes.

## Abstract (precis)

Fusarium udum F02845 is a destructive fungal pathogen which causes pigeonpea (Cajanus cajan L. Millspaugh) wilt.

## ANNOUNCEMENT

Wilt caused by Fusarium udum F02845 is one of the most important diseases of pigeonpea. The disease has been reported from several countries, including India, Bangladesh, Mauritius, Ghana, Kenya, Malawi, Tanzania, Uganda, Indonesia, Thailand, and Trinidad (1). The fungus causing wilt can survive on infected plant debris in soil for about 2 to 3 years, and it is responsible for causing 16 to 47% yield loss under favorable environmental conditions (2). At present, chemical and biological disease management strategies for containing this fungus are not very effective; therefore, genome sequencing can divulge virulence-related genes for better understanding of host-pathogen interactions. So far, molecular investigations on the pathogenicity of F. udum have not been performed. Here, we describe the first draft genome sequence to assist further genome-based examination of F. udum and its host-pathogen interactions.

The isolate of F. udum (F02845) was collected from a pigeonpea stem displaying pronounced symptoms of wilt disease in the year 2010 from Bahraich (27°34′31.4″ N, 81°35′33.6″ E), Uttar Pradesh, India. The fungal isolate was cultivated on potato dextrose agar (PDA), and total genomic DNA was extracted with cetyl‐trimethylammonium bromide (CTAB), as described by Kumar et al. (3).

The draft genome was sequenced with the Illumina NextSeq sequencer, using a HiSeq 2000 platform for paired-end reads. A TruSeq Nano DNA library kit (Illumina) was used for sequencing-library preparation. A library of 28.08 million paired-end reads (read length, 101 bp; insert size, 433 bp) of 28.36 Gb total size was generated. The Next-Generation Sequencing Quality Control (NGS QC) toolkit version 2.3 (4) was used to filter high-quality data (at a Phred score of 20), and 24.99 million high-quality reads were obtained. Primary genome assembly was done using the program Velvet version 1.2.10 (5) with a kmer length of 81. The assembled genome was 56,750,279 bp in length, with an *N*_50_ value of ∼0.08 Mb, resulting in 10,427 contigs. The scaffolding of primarily assembled data was done using SSPACE version 3.0 (6), resulting in 2,634 scaffolds at a maximum link ratio of ≥0.5. The maximum and average scaffold lengths were ∼0.7 Mb and ∼0.21 Mb, respectively. After removing scaffolds that were less than 200 bp by using CONTIGuator 2.7 (7), the final assembly consisted of 712 scaffolds with a genome size of 56,381,318 bp (42.44% G+C content). Interspersed repetitive elements and low-complexity DNA sequences were masked using RepeatMasker version 4 (8), followed by rRNA and tRNA prediction using RNAmmer v1.2 (9) and tRNAscan-SE v1.3.1 (10), respectively. Prediction of protein-coding genes was performed using GeneMark-ES fungal version 2 (11). Functional classification of the predicted proteins was done using the EuKaryotic Orthologous Groups of proteins database (KOG) (12), while motifs and protein domains were predicted with InterProScan v5 (13). The dbCAN database was used to predict carbohydrate-active enzymes (14). Overall, the whole genome encompasses 11,829 protein-coding genes, 296 tRNAs, and 53 rRNAs. A total of 8,928 genes were categorized into functional groups using the KOG database (11). Furthermore, the genome contained 1,439 signal peptides, 15,649 transmembrane helices, 2,858 carbohydrate-active enzymes (CAZy), 3,682 transporter genes, and 1,060 putative pathogenicity genes.

### Data availability.

The present draft genome assembly has been deposited in the NCBI repository under GenBank accession number NIFK00000000 and assembly accession number GCA_002194535 (BioProject number PRJNA385264). Short-read data have been submitted to the SRA under NCBI accession number SRP157084.
